# Magnetic-based Soft Tactile Sensors with Deformable Continuous Force Transfer Medium for Resolving Contact Locations in Robotic Grasping and Manipulation

**DOI:** 10.3390/s19224925

**Published:** 2019-11-12

**Authors:** Alireza Mohammadi, Yangmengfei Xu, Ying Tan, Peter Choong, Denny Oetomo

**Affiliations:** 1 Department of Mechanical Engineering, The University of Melbourne, Parkville, VIC 3040, Australia; yangmengfeix@student.unimelb.edu.au (Y.X.); yingt@unimelb.edu.au (Y.T.); doetomo@unimelb.edu.au (D.O.); 2 Australian Research Council Centre of Excellence for Electromaterials Science, Wollongong, NSW 2500, Australia; pchoong@unimelb.edu.au; 3 Department of Surgery, University of Melbourne, St Vincent’s Hospital, Fitzroy, VIC 3065, Australia

**Keywords:** contact location in tactile sensor, soft tactile sensors, deformable continuous force transfer medium, array of discrete tactile sensors, soft robotics, robotic hand and gripper

## Abstract

The resolution of contact location is important in many applications in robotics and automation. This is generally done by using an array of contact or tactile receptors, which increases cost and complexity as the required resolution or area is increased. Tactile sensors have also been developed using a continuous deformable medium between the contact and the receptors, which allows few receptors to interpolate the information among them, avoiding the weakness highlighted in the former approach. The latter is generally used to measure contact force intensity or magnitude but rarely used to identify the contact locations. This paper presents a systematic design and characterisation procedure for magnetic-based soft tactile sensors (utilizing the latter approach with the deformable contact medium) with the goal of locating the contact force location. This systematic procedure provides conditions under which design parameters can be selected, supported by a selected machine learning algorithm, to achieve the desired performance of the tactile sensor in identifying the contact location. An illustrative example, which combines a particular sensor configuration (magnetic hall effect sensor as the receptor, a selected continuous medium and a selected sensing resolution) and a specific data-driven algorithm, is used to illustrate the proposed design procedure. The results of the illustrative example design demonstrates the efficacy of the proposed design procedure and the proposed sensing strategy in identifying a contact location. The resulting sensor is also tested on a robotic hand (Allegro Hand, SimLab Co) to demonstrate its application in real-world scenarios.

## 1. Introduction

The resolution of contact locations is an important feature required in many robotics applications involving grasping and manipulation [1,2]. In the context of robotic grasping and manipulation, the knowledge of the location of contact with the object (i.e., where contact force is applied by the object to the robotic hand/gripper) is very important in validating the grip configuration, evaluating the satisfaction of wrench closure condition, allowing the robot to adjust its grip on an object to reject disturbances and to ensure that the object remains within the manipulable part of the hand/fingers during manipulation [2,3,4,5,6,7,8,9]. Due to the complexity of the manipulation tasks, model-based methods utilising force/torque sensors to detect the location of the applied contact force have not been shown to be robust [10,11]. It is, therefore, necessary for tactile sensors to be developed to identify locally the contact locations of the object with the robot/gripper.

There is a wide range of different tactile sensors depending on their modality to obtain contact information [12]. In terms of the medium of detection mechanism for contact localisation, tactile sensing methods may be classified into two main approaches: (1) using an array of discrete individual tactile receptors, where the contact location is identified by which individual receptor is triggered, and (2) using a continuous medium which deforms due to the contact and alters the transmitted signal to the sensing mechanism.

In the array of discrete sensor approach, each individual sensor form an independent receptor and its signal is individually processed. In this type of sensors, each receptor unit only takes charge of contact detection in one specific region and ideally, one can neither influence the results of the other sensor units nor be affected by others. The array of discrete sensor can be realised using one of many sensing modality, for example: the strain gauge sensor skin [13,14], array of capacitive sensors [15], tactile array based on barometric measurements [16] and magnetic-based soft distributed skin [17]. These sensors have been successfully used in many robotic hands for manipulation tasks. The main advantage of this method is its ability to detect multiple contact points simultaneously. The main weakness with the array-based tactile sensors is that the number of individual sensors increases for a higher spatial resolution, which introduces a physical limit to the resolution of the contact location, increased cross-talk between sensing elements when packed closer to each other and relatively complex circuitry [12]. The increase in the number of sensors for higher resolution also means increased cost. In addition, most of the array-based tactile sensors are manufactured in a flexible flat shape which cannot be easily formed into the desired shapes.

In the second approach, the sensor mechanism consists of a continuous force transfer medium, a signal source and the corresponding signal receptors whose responses vary as a function of the deformation of the medium under the contact force. The receptor is usually embedded on the rigid member of the robotic hand or finger, in physical contact with the deformable medium but does not move with the deformable medium. Upon contact with the robotic gripper or finger, the contact force causes deformation on the medium relative to the receptors and causes a change in the signal that is picked up by the receptor. For example, OptoForce [18] is a commercial tactile sensor based on this concept. An elastic semi-sphere dome is used as force transmission medium, with a light source and receptor under the dome. When force is applied to the deformable medium, the surface deforms and causes variation in the reflected light beams which are detected with infrared sensors as receptors.

In contrast to many discrete tactile sensors needed, the sensors with continuous medium require fewer receptors and processing units at the cost of requiring more complex algorithms to achieve the contact force localisation. Hence, the resolution is not restricted by the size or the quantity of the receptor units. Under such a situation, the number of receptor units is a function of the number of independent readings required to obtain a unique solution to solve for the unknowns, which in our case is the location of the contact point expressed in a given coordinate frame. Such a setting also provides higher flexibility in design of the desired shape, curvature and volume of the sensor for different robotic applications, often achievable through the moulding of the deformation medium to the intended shape. As expected, it is challenging for such a sensor to discern more than one contact force (at one contact location) per individual piece of continuous medium. This is regarded as an acceptable limitation in this paper. For example, for a robotic grasping problem, most objects are large enough to result only in one contact point per finger segment of the robotic hand/gripper.

In the proposed approach of using continuous deformable medium, there are two widely used signal modalities: optical and magnetic. Applied force causes the light transmission medium to deform resulting in a variation in the reflected light beams [19] which allows the applied contact force to be quantified. OptoForce, which has been set to measure the magnitude of the contact force (in 3D) but not the contact location, is an optical tactile sensor. TacTip [20] is another example of an optical sensor that operates using an embedded camera to track the deformations of the pins on the inside surface of its deformable dome-shaped membrane. This sensor can provide both the contact force location and the magnitude of the contact force. A variety of TacTip sensors are developed for robotic applications [21], however, these sensors require rigid camera systems that would be difficult to integrate in each phalange of multi-fingered robotic hands, especially in soft robotic hands [22,23]. Vision-based sensing also conventionally requires relatively higher computational costs.

Magnetic-based tactile sensor, which consists of permanent magnets, Hall effect sensors, and a soft medium is another example of a tactile sensor with continuous medium. Deformations on the soft medium due to the applied force will result in displacement of the permanent magnet which can be measured by magnetic field sensors as receptors [24,25]. These sensors are compact, low cost, highly sensitive and easy to integrate in robotic systems as their soft medium can be designed in different shape and size [12,19,26]. Most of current magnetic-based soft tactile sensors are aiming to provide the force measurement at a priori known contact location and the sensor is calibrated for that point of contact. For instance, in [27] a pyramid shape of the force transfer medium is used to ensure that the sensor contacts the objects with its tip. As a result, these sensor designs cannot provide the contact force location on the sensor.

This paper focuses on the systematic design and characterisation procedure when the location of the contact force is needed for a large class of magnetic-based tactile sensors with a continuous force-transfer deformation medium. We consider a general class of configurations of sensors including the arrangement of the multiple permanent magnets (as the source of the signal to be deformed upon contact) and Hall effect sensors (as receptors), general shape, size and stiffness of the soft medium. Given the desired configuration of the sensor, the location of the contact force can be calculated. Such a procedure requires (1) a reliable mapping between the contact force location and the resulting displacement of the deformable medium (within which a set of magnetic source in the form of permanent magnets are embedded) and (2) the mapping between the resultant magnetic field (and its changes due to the contact force) and measurements from Hall effect sensors serving as the receptors. Both mappings are highly nonlinear and sensitive to the configuration of the sensors. While the equation of the resultant magnetic field is readily available, a model-based method is neither practical nor robust in determining the accurate deformation of the continuous medium. The alternative approach is using data-driven methods [27,28] which do not require the model of deformable medium and can provide direct relation between the magnetic field variation from the contact force and Hall effect sensor measurements.

The objective of this paper is to propose a systematic design procedure for the design of the soft tactile sensor in locating the contact force location. This design and characterisation procedure provides conditions under which one can combine different sensor configurations, supported by a selected (basic) machine learning algorithm to achieve the desired performance of the tactile sensor. The sensing performance is given in the accuracy of contact force location detection for a given shape and size of the deformable force transfer medium and a given spatial resolution of the sensing location. Moreover, the conditions provide a systematic approach in finding the minimum number of required permanent magnets and Hall effect sensors and optimum stiffness of the soft medium. An illustrative example, which combines a particular sensor configuration and a specific data-driven algorithm, is used to illustrate the proposed design procedure.

However, the relation between the external contact force and the resulting displacement of the deformable medium (and hence, that of the permanent magnet(s)) is challenging to obtain. Finite element analysis (FEA) has been employed to describe this relation. However, these models are not suited for real-time computation. The alternative approach is using data-driven models. These models do not require the model of deformable medium and can provide a direct relation between the magnetic field variation due to the contact force and Hall effect sensor measurements. Nevertheless, the sensor design in these papers are presented for a particular shape and size of the sensor and particular arrangement of the permanent magnets and Hall effect sensors.

## 2. The Proposed Sensor Design Procedure

### 2.1. Sensor Configuration

**Figure 1:**
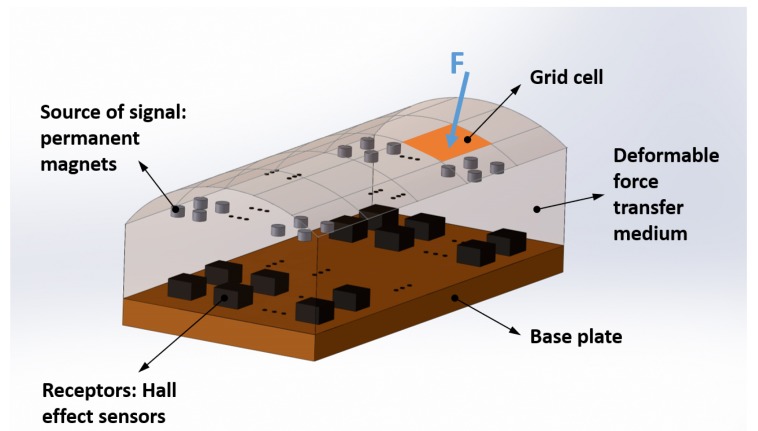
The general case configuration of the proposed tactile sensor with *n* permanent magnets as the sources of the signal and *m* Hall effect sensors as the signal receptors, embedded in the deformable force transfer medium. The intended spatial resolutions of the contact point localisation procedure are defined by the size of the grid cell.

Figure 1 shows the general configuration of the proposed sensor. It consists of multiple permanent magnets as sources of a magnetic field, multiple Hall effect sensors as the receptors to measure the magnetic field, and a deformable medium for force transfer. The applied force *F* will result in the deformation of the medium and a change in the pose (position and orientation) of the permanent magnets embedded in it.

We assume that the following desired performance characteristics and physical properties are given in advance as design requirements:

- the shape and size of the sensor unit (the base plate and the deformable medium)
- the contact surface area on the medium (*A* [mm${}^{2}$])
- the desired spatial resolution of the identified contact location, defined as the number of grid cells (or the dimension of the grid cell) as shown in Figure 1 (*k*)
- the minimum detectable applied force on sensor ($F_{min}\left[N\right]$)
- the permanent magnet properties including size and magnetic moment (m [Nm/T])
- and the specifications of the Hall effect sensor, such as the sensitivity ($S_{H}\left[T\right]$), saturation ($B_{sat}\left[T\right]$), minimum measurable magnetic field ($B_{min}\left[T\right]$).

Based on the above information, it is desired to find the conditions under which the minimum number of permanent magnets required, the optimum stiffness of the deformable medium and the minimum number of Hall effect sensors required, can be obtained. The knowledge obtained is then used to resolve the contact location of the applied force on the sensor through a data-driven contact localisation algorithms.

It should be noted that in the proposed procedure, it is assumed that only one contact point is applied to one sensor unit. We justify the assumption by the argument that when used as the tactile sensor for a robotic hand to grasp objects, the variations in the surface topology and shape of the common objects are generally large enough to only result in one contact location per sensor unit, even if the contact area could be large to be considered as a point contact.

It is initially defined that *n* permanent magnets and *m* Hall effect sensors are employed. The magnetic field density of the permanent magnets can be expressed as [29]:

(1) $$B_{PM}=\frac{\mu_{0}}{4\pi }\left(\frac{3\left(m.\hat{r}\right)\hat{r}-m}{\left|r\right|}\right),$$

where $\mu_{0}=4\pi \times 10^{-7}$ is the permeability of free space, m is the magnetic moment of the permanent magnet, r is the distance from center of permanent magnet and $\hat{r}$ is the unit vector in the direction of r. The superposition of the magnetic field of the permanent magnets measured by a Hall effect sensor ($H_{j}$) is:

(2) $$B_{@H_{j}}=\sum_{i=1}^{n}B_{PM}\left(r_{ij}\right),$$

where j∈[1,m], $r_{ij}$ is the distance of permanent magnet *i* from the Hall effect sensor *j* and it is a function of the force-transfer medium shape and size. An applied force to the sensor unit would deform the medium, in which the permanent magnets are embedded, which in turn causes a change in the magnetic field measured at the Hall effect sensors.

**Condition** **1.** *If no force is applied on the sensor, then there exists one* j∈[1,m] *such that:*

$$\begin{array}{cc} B_{min}<\left|B_{@H_{j}}\right| & <B_{sat}. \end{array}$$

This condition states that the superposition of magnetic field of all permanent magnets is greater than minimum measurable magnetic field of at least one Hall effect sensor and less than the saturation value of that sensor.

**Condition** **2.** *If*$F_{min}$*is applied in each of k grid cells of the sensor, then for all cells of the sensor surface*, 1,…,k, *there exists one*j∈[1,m]*such that:*

$$\begin{array}{cc} \Delta B_{@H_{j}} & >S_{H}. \end{array}$$

This condition guarantees that when the minimum force is applied to each grid cell, the variation of the magnetic field is more than the sensor sensitivity. This condition is needed to ensure that at least one Hall effect sensor will be affected by the applied contact force.

**Remark** **1.** *It should be noted that using a large number of permanent magnets does not necessarily result in satisfaction of condition 1 as superposition of the magnetic field of permanent magnets can cancel out the effect of each other.*

**Remark** **2.** *Conditions 1 and 2 are necessary but not sufficient for the desired performance of the sensor in locating the contact location since the sensor performance is also related to the machine learning algorithm used to provide the mapping between the Hall effect sensor readings and the applied force, which are highly non-linear.*

### 2.2. Contact Localisation Algorithm

After the design of sensor configuration with the desired number of permanent magnets and Hall effect sensors based on required conditions, we need a contact localisation algorithm for the specific sensor that results from the design exercise. If a data-driven algorithm is used, then it is required to find the relationship between the external force contact location and Hall effect sensor measurements. In other words, the input to this data-driven algorithm is the Hall effect sensor measurements and the output is the contact point location (which grid cell) on the surface of the sensor. In order to collect the required data for such an algorithm, we divide the surface of the force transfer medium to a number of grid cells depending on the desired resolution of the sensor and label them from 1 to *k*. Then, contact forces with different magnitudes are applied to the sensor and collect the corresponding measured magnetic fields from Hall effect sensors.

After collecting the required data, one of many classification algorithms can be employed for training the data-driven algorithm. Depending on the selected algorithm, the parameters of the algorithm can be tuned to a desired performance. This will be explained with an example in Section 3.2.

## 3. Illustrative Example and Experimental Validation

In this section, an example configuration of the proposed tactile sensor is designed, fabricated and used to illustrate the evaluation of the Conditions 1 and 2 and implementation of the contact location resolution algorithms.

### 3.1. Sensor Design and Fabrication

**Figure 2:**
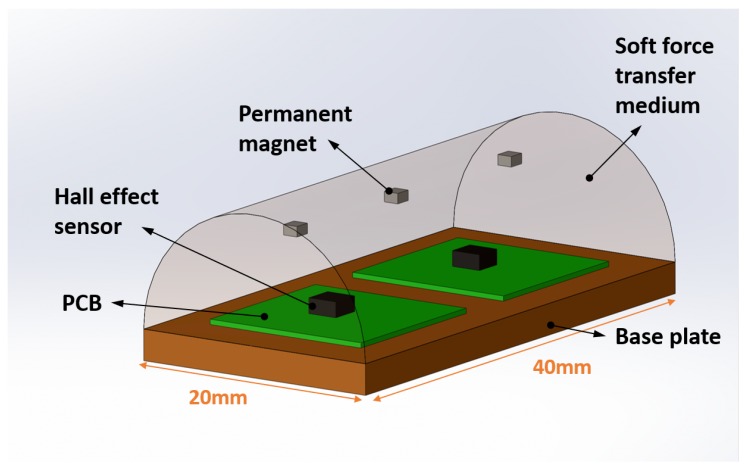
A specific design of the soft tactile sensor with three permanent magnets and two Hall effect sensors embedded inside a semi-cylinder force transfer medium made of Polyurethane (VytaFlex 20).

Figure 2 shows the schematic arrangement of three permanent magnets and two Hall effect sensors in the soft tactile sensor. The deformable force-transfer medium was considered as a semi-cylinder constructed over a 40 mm × 20 mm base plate as shown in Figure 2. The permanent magnets were neodymium block magnets with a size of 2 × 2 × 1 mm height and magnetic moment of 1.4 Nm/T. These magnets were placed at the middle axis of the sensor with 10 mm distance from each other and 10mm from the sensor base plate. The Hall effect sensors are MLX90393 tri-axis magnetic sensors from Melexis Inc. (Leper, Belgium) providing magnetic field measurements in three axes thorough I2C fast mode protocol. The Hall effect sensors were placed in 20 mm distance from each other and 10 mm from two ends of the cylinder.

**Figure 3:**
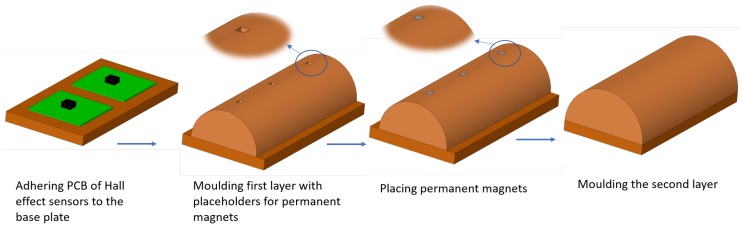
Sensor fabrication procedure.

The sensor is fabricated using a two-stage moulding process as shown in Figure 3. At first stage, the PCB of Hall effect sensors was adhered to the base plate and then assembled with a 3D printed mould which included an extruded negative of the permanent magnets shape to create a placeholder for the magnets. Polyurethane rubber (VytaFlex20 from Smooth-On Inc. (Macungie, PA, USA)) with Shore hardness of 20 A and tensile strength of 200 psi) was then poured into the mould and de-moulded after curing. At the second stage, the magnets are placed in their placeholders and the second 3D printed mould is assembled and the second layer of the polyurethane rubber is injected in the mould.

Given this specific size and shape of the sensor and the stiffness of the deformable medium, condition 1 was evaluated for this particular configuration.

**Figure 4:**
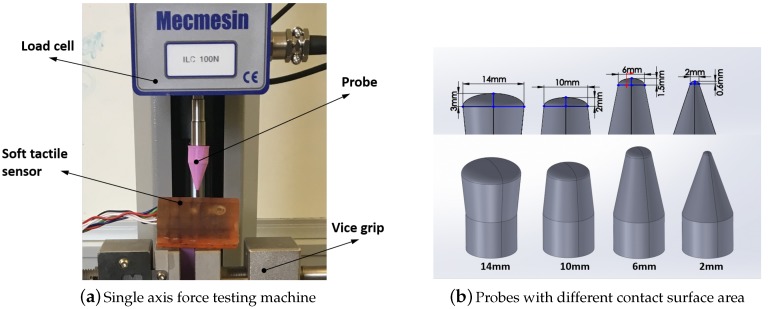
Experimental setup for data collection using a single-axis force testing machine to apply desired force on the sensor with different probes.

To do so, the magnetic field readings in Hall effect sensors 1 and 2 were obtained when no force was exerted on the senor. The values were found to be

$$\begin{array}{c} 10\;\mu T\;<\;|B_{@H1}|\;=\;3300\;\mu T\;<50\;\mathrm{mT},\\ 10\;\mu T\;<\;|B_{@H2}|\;=\;3650\;\mu T\;<\;50\;\mathrm{mT}, \end{array}$$

therefore, it satisfies Condition 1. To verify Condition 2, the maximum desired resolution of the sensor surface was assumed to be a grid with 25 cells as shown in Figure 4. A contact force was exerted on each of these cells and the minimum force that results in variation of the magnetic field which can be detected with one of two Hall effect sensors was measured. As a result, a matrix with 25 values representing the grid cell is produced as:

$$F_{min}=\left[\begin{array}{ccccc} 1 & 0.5 & 0.5 & 0.5 & 0.5\\ 0.5 & 1 & 0.5 & 0.5 & 0.5\\ 0.5 & 0.5 & 0.5 & 0.5 & 0.5\\ 0.5 & 1 & 0.5 & 0.5 & 0.5\\ 1.5 & 1 & 0.5 & 1 & 1.5 \end{array}\right]$$

Therefore, the minimum contact force required to satisfy Condition 2 is the maximum value in the $F_{min}$ matrix which is $max(F_{min})=1.5N$. If it is desired to discern the contact location of forces with lower magnitude, the following parameters should be altered in the sensor design:

- If $max(F_{min})$ is high, we need to change the stiffness of the soft force transfer medium in order to have a larger displacement of permanent magnets;
- If a few number of cells have high $F_{min}$ value, then we need to add more permanent magnets close to those cells;
- If a block of adjacent cells have high $F_{min}$ value, then we need to add extra Hall effect sensor(s) close to those cells.

Since both conditions are satisfied, it can be concluded that by using an appropriate classification algorithm, the desired performance of the sensor can be achieved. To this end, a sufficient amount of data needs to be collected for the training and validation of the classification algorithm selected.

### 3.2. KNN Algorithm for Contact Localisation

In this paper, K-nearest neighbours (KNN) algorithm was selected, which is one of the most common classification algorithms. It should be noted that the proposed framework is not restricted to a specific classification algorithm and depending on the amount of collected data, speed of algorithm, etc., an appropriate algorithm can be employed. In the KNN classifier, for each set of training data one label is assigned. Every time a new datum point needs to be classified, the algorithm will select K closest samples in terms of feature similarity in the training data set. Finally, these new data points will be classified through a majority vote among its nearest K neighbours. As this method is based on the raw data, it allows the existence of noise in the training data, which can account for small dynamics complexities in the sensor such as the hysteresis of the deformable medium. The other advantage of the KNN algorithm which makes it suitable for our application is that it is a non-parametric technique and does not make any assumptions on the underlying data distribution such as linear relation. Therefore, KNN is one of the first choices for a classification study when there is little or no prior knowledge about the distribution of the measured data.

### 3.3. Data Collection Method

To collect the required data, the experiments were conducted using a single-axis force testing machine (Mecmesin MultiTest 2.5i Testing Centre 2500 with 100N load cell) to apply an external force on the surface of the sensor as shown in Figure 4. The sensor was fixed using a vice and a 3D printed probe was used to apply the force over different grid cells of the sensor. Each datum point is an array of the magnetic field values of the two Hall effect sensors, $\left(B_{x1},B_{y1},B_{z1},B_{x2},B_{y2},B_{z2}\right)$. The data were transferred to a PC using an Arduino Uno microcontroller.

In order to evaluate the performance of the contact localisation algorithm in different real-world scenarios, we will collect three sets of data to investigate the effect of the following three cases:

- Sensor resolution: to investigate the accuracy of the KNN contact point localisation algorithm in different resolutions of the sensor, the surface of the sensor is divided to 6, 15 and 25 grid cells. This results in 6, 15 and 25 classes in the KNN algorithm, where each grid cell corresponds to a single class. The force is applied in the normal direction on each grid cell with magnitudes varying from 1.5 N to 10 N and $\left(B_{x1},B_{y1},B_{z1},B_{x2},B_{y2},B_{z2}\right)$ is obtained. This process is repeated five times for each cell. For resolutions 6, 15 and 25, the total number of sample points collected are 2400, 6000 and 10,000, respectively. The total collected data is partitioned randomly in 20–80%, 50–50% and 80–20% ratio for training and testing. In random sampling, every observation in the main data set has an equal probability of being selected for the partition data set. The performance of the algorithm is investigated in these three training-testing ratios. We also investigate the effect of different K values (K = 1, 5, 10) in the accuracy of the contact point localisation.
- Contact forces in different directions: the previous set of data are collected where the contact forces are normal to the surface of the sensor. Although, in real-world scenarios, forces can be applied in any directions. Therefore, in this case, forces are applied to each grid cell in random directions. These forces, however, are confined inside a cone with 45 degrees angle with the cell surface. For the data collection for this case, the sensor resolution 15 and 3D printed probes with a tip diameter of 2 mm and 6 mm are used. The KNN algorithm was set to 50% training data and K = 5.
- Contact surface area: to explore the effect of contact surface area on the performance of the sensor, four probes with different tip sizes (2 mm, 6 mm, 10 mm and 14 mm) as shown in Figure 4 are used to collect the data. This represents the variations in the contact surface area that may result in a practical implementation. For example, when the tactile sensor is mounted on a robotic hand and grasp an object such as a mug, where the contact between the sensor and the mug is not just a point, but a surface area. The data collection procedure was similar to the previous data set collection, in different directions with sensor resolution 15, 50% training data and K = 5.

## 4. Results and Discussion

In this section, the performance of the sensor is investigated for using a combination of the proposed design configuration in Section 3.1 and the KNN classification algorithm in Section 3.2. The performance of the sensor is reported in terms of the accuracy of contact localisation. When considering sensors with different resolutions (different grid cell), the average Euclidean error is also considered. Euclidean error (EE) [28] is a metric to weigh the cost associated with each misclassified class by considering its 2D Euclidean distance from its true response class.

### 4.1. Sensor Resolution

**Figure 5:**
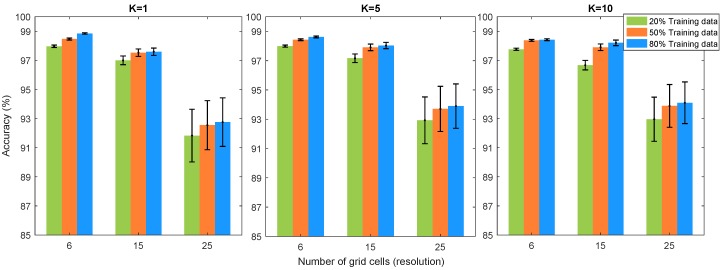
Accuracy of contact force location detection in terms of surface resolution, size of training data and K values of K-nearest neighbours (KNN) algorithm.

The results of contact localisation accuracy in different resolutions of the sensor are shown in Figure 5 for different training data percentage and different K values of the KNN algorithm. The results show that the training data percentage and K value of the KNN algorithm do not significantly affect the accuracy of contact localisation. Therefore, we can use 20% training data with K = 1, which will result in a significant reduction in the training and response time. The minimum accuracy of all different combinations is more than 92%. As expected, the localisation accuracy of different resolutions decreases with increasing the resolution although this reduction is in an acceptable range (maximum 7% reduction). The localisation accuracy in resolutions 6 and 15 are similar.

**Figure 6:**
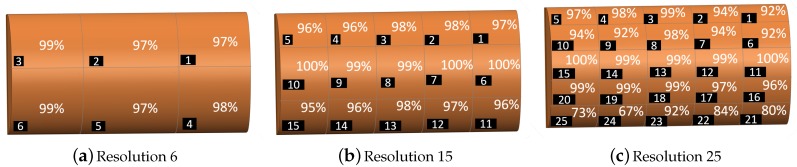
Accuracy of contact force location detection for each cell of sensor grid at different resolutions with K = 5 and 50% training data.

In order to determine which cells of the sensor contribute more to the misclassification percentage, localisation accuracy of each cell of the sensor in different resolutions are shown in Figure 6 for the case of K = 5 and 50% training data. The results show that the cells far from permanent magnets and Hall effect sensors (e.g., cell 21 and 25 in the resolution = 25) had lower accuracy and the middle axis of the sensor on which permanent magnets are placed had the highest accuracy. Therefore, with adding extra permanent magnets close to the cells with lower accuracy we can get higher overall detection accuracy.

**Figure 7:**
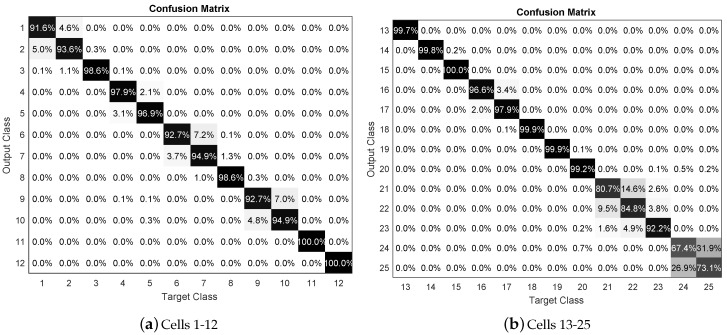
Accuracy of contact force location detection for resolution of 25 cells.

Moreover, KNN classifier confusion matrix is shown in Figure 7 for the 25 grid cells resolution with K = 5 and 50% training data. The diagonal of the matrix shows the percentage of the correct classification and off-diagonal elements show misclassifications (false positive in the upper-right and false negative in the lower left).

**Figure 8:**
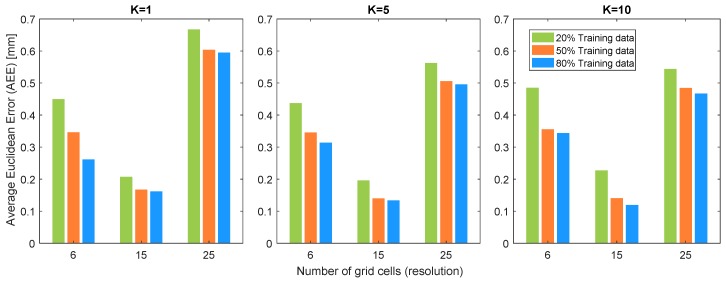
Average Euclidean error (AEE) in terms of surface resolution, size of training data and K values of KNN algorithm.

In order to find the Euclidean distance between different classes of the sensor, the 3D grid cells of the sensor surface are projected to the 2D base of the sensor and the distance between the centre of each cell from the centre of the other cell is considered as Euclidean distance. Average Euclidean error (AEE) is defined as the accumulation of all the EEs divided by the number of classes (grid cells of the sensor). Figure 8 depicts the AEE results for different training data percentage, different resolution of the sensor and different K values of the KNN algorithm. Since standard deviations are less than 0.01 mm for all results, they have not been shown in the bar plots. AEE results show that increasing the training data percentage will result in a slight decrease in the AEE. Similarly, increasing the K value in KNN algorithm will decrease the AEE marginally. The interesting point in the results of AEE is related to the resolution of the sensor where the 15 grid cell resolution has the minimum AEE in comparison to resolution 6 and 25. This is due to the trade-off between grid cell size and the accuracy of the contact localisation. In other words, the misclassified grid cell in 25 grid cell resolution will contribute smaller penalty values to AEE compared to those in 6 and 15 grid cell accuracy, but it has lower contact localisation accuracy as well. Therefore, this trade-off will result in minimum AEE in resolution 15. This is also in agreement with the results of contact localisation accuracy in the previous section whereby resolution 15 has the optimum performance in having a good trade-off between resolution and contact localisation accuracy.

### 4.2. Contact Forces in Different Directions

**Figure 9:**
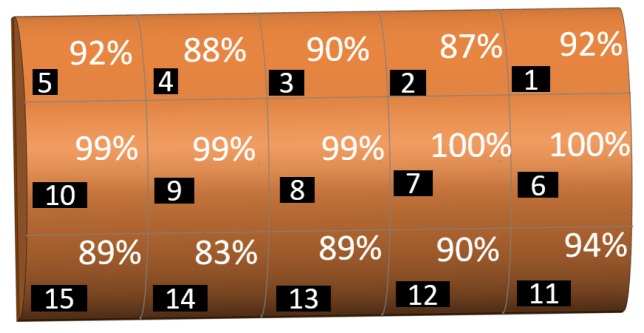
Contact localisation accuracy for individual grid cells of the sensor with contact forces in normal and non-normal directions using 2 mm and 6 mm probes.

In this case, data sets were collected with forces applied in random contact directions. The data is partitioned for training of the classifier and to validate the resulting classifier. Two probe sizes used were 2 mm and 6 mm. When the data set was collected using 2 mm probe for both training and validation, an accuracy of 92% was achieved. Figure 9 shows the contact localisation accuracy with 2 mm probe for individual grid cells of the sensor. When 6 mm probe was used for both training and validation, an accuracy of 96% was achieved. When both 2 mm and 6 mm probes were used (randomly distributed) in the same data set for both training and validation, an average accuracy of 93% was achieved. Therefore, given a reasonable amount of data, the data-driven classifier approach was found to provide acceptable performance in the resolution of the contact location when dealing with arbitrary direction of the applied force. Using other advanced learning algorithms or regression methods can potentially reduce the amount of data needed to be collected.

### 4.3. Contact Surface Area

The experiments are conducted with contact forces in the normal direction with four different sizes of the probes as shown in Figure 4. The surface of the larger probes (diameters 10 mm and 14 mm) does not fully touch the surface of the sensor when applied in a non-normal direction. As a result, the effect of contact surface area on the accuracy of the contact localisation is only investigated in normal direction. The results are shown in Table 1. In the cases where the same probe is used for training and testing data, the accuracy of contact localisation is more than 98% (the accuracy percentage of the model on the diameter of the Table 1). This indicates that irrespective of the probe sizes, the accuracy of the contact localisation is highly dependent on the consistency of the data used for training and testing the model.

Additionally, Table 1 shows relatively high accuracy for the cases when different probe sizes were used for training and for testing. The performance consistent excludes that for the 2 mm probe data. In this case, deformations of the soft force transfer medium are small due to the small probe diameter (in comparison to other probes) which results in small variations in the permanent magnet’s displacement and hence magnetic field measurements. Therefore, the behaviour of the 2 mm probe cannot be grouped with other probes. In other words, there is a lower limit to the size of the probe used in either training or testing data.

Finally, if we train and test the classifier with all the observations from all probe sizes, the overall accuracy is 97%. This shows that the accuracy of the contact localisation when trained and tested with the data collected from all probes (the integrated dataset) is still similar to those with the individual probe (subset of the integrated dataset).

## 5. Demonstration on Allegro Robotic Hand

**Figure 10:**
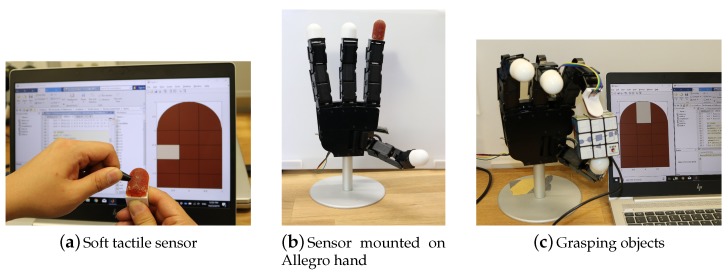
Demonstration of the proposed tactile sensor application in detection of the contact location of an object with a robotic hand.

To demonstrate the application of the proposed tactile sensor design in real-world scenarios, we mounted the sensor on the Allegro robotic hand (SimLab Co., Seoul, Korea). One of the fingertips of the hand is replaced by our soft tactile sensor as shown in Figure 10. The sensor has the same configuration of the permanent magnets and Hall effect sensors as described in Section 3.1, the only difference is a slight change in the shape of the soft force transfer medium in order to make it similar to the other fingertips of the hand. This change also facilitates the grasping ability of the finger. The procedure of data collection with forces in normal and non-normal directions and with different probe sizes was performed for this sensor while the resolution is 15.

We used the Allegro hand with integrated soft tactile sensor to grasp three objects (a Rubik’s cube, a 3D printed sphere and a wireless mouse) and visually checked the correctness of the contact location detection. Each object has been grasped 50 times and the accuracy of the contact location prediction for the Rubik’s cube, 3D printed sphere and wireless mouse were 94%, 96% and 94%, respectively. A video showing real-time contact point detection is available as Supplementary Materials to this paper.

## 6. Conclusions

In this paper, a systematic design and characterisation procedure for magnetic-based soft tactile sensors with continuous force transfer medium is presented with the purpose of resolving the location of the contact force. The required conditions for the desired configuration of the sensor are presented. An illustrative example of the sensor configuration with three permanents magnets and three Hall effect sensors and classification method of KNN algorithm demonstrated the minimum accuracy of 92% in contact force localisation, given different resolutions of the sensor, different contact surface areas and with forces applied in random contact directions. 

## Figures and Tables

**Table 1:** Contact point localisation accuracy using probes with different sizes.

Training Dataset	Testing Dataset			
	2 mm Probe	6 mm Probe	10 mm Probe	14 mm Probe
2 mm probe	98%	63%	64%	55%
6 mm probe	45%	98%	83%	73%
10 mm probe	49%	80%	99%	92%
14 mm probe	44%	71%	95%	99%
All probes	97%			

## Supplementary Materials

The following are available online at https://www.mdpi.com/1424-8220/19/22/4925/s1.
